# Trends in Preterm Births in Italy and Maternal Risk Factors in 2018–2022—A Registry-Based Study

**DOI:** 10.3390/children12030257

**Published:** 2025-02-20

**Authors:** Franca Rusconi, Martina Pacifici, Anna Maria Nannavecchia, Sonia Brescianini, Teresa Spadea, Pietro Buono, Michele Gobbato, Olivia Leoni, Eva Papa, Enrica Perrone, Riccardo Pertile, Arianna Polo, Monia Puglia, Raffaella Rusciani, Elisa Eleonora Tavormina, Laura Visonà Dalla Pozza, Luigi Gagliardi

**Affiliations:** 1 Department of Mother and Child Health, Azienda USL Toscana Nord Ovest, 56121 Pisa, Italy; luigi.gagliardi@uslnordovest.toscana.it; 2 Unit of Epidemiology, Regional Health Agency of Tuscany, 50141 Florence, Italy; martina.pacifici@ars.toscana.it (M.P.); monia.puglia@ars.toscana.it (M.P.); 3 Department of Epidemiology and Care Intelligence, Strategic Regional Agency for Health and Social Affairs—AReSS Puglia, 70121 Bari, Italy; a.nannavecchia@aress.regione.puglia.it; 4 Centre for Behavioral Sciences and Mental Health, Istituto Superiore di Sanità, 00161 Rome, Italy; sonia.brescianini@iss.it; 5 Department of Epidemiology, ASL TO3 Piedmont Region, Collegno, 10093 Turin, Italy; teresa.spadea@epi.piemonte.it (T.S.); raffaella.rusciani@epi.piemonte.it (R.R.); 6 Department of Maternal and Child Health, General Directorate for Health, Campania Region, 80131 Naples, Italy; pietro.buono2@regione.campania.it; 7 Pianificazione Programmazione e Controllo Direzionale, Azienda Regionale di Coordinamento per la Salute del Friuli-Venezia Giulia, 33100 Udine, Italy; michele.gobbato@arcs.sanita.fvg.it; 8 Epidemiologic Observatory, Welfare General Directorate, Lombardy Region, 20124 Milan, Italy; olivia_leoni@regione.lombardia.it; 9 Observatory for Health, Health Department Province of Bolzano, 39100 Bolzano, Italy; eva.papa@provincia.bz.it; 10 Unit of Primary Care, Regional Health Authority of Emilia-Romagna, 40127 Bologna, Italy; enrica.perrone@regione.emilia-romagna.it; 11 Department of Clinical and Evaluative Epidemiology, Health Service of Trento, 38123 Trento, Italy; riccardo.pertile@apss.tn.it; 12 Area Rete Ospedaliera, Lazio Regional Authority, 00145 Rome, Italy; apolo@regione.lazio.it; 13 Institute for Biomedical Research and Innovation, National Research Council, 90145 Palermo, Italy; e.tavormina@villasofia.it; 14 Department of Health Activities and Epidemiological Observatory, Regional Health Authority, Sicilian Region, 90133 Palermo, Italy; 15 Birth Registry, Coordinating Centre for Rare Diseases, Veneto Region, 35100 Padua, Italy; laura.visonadallapozza@regione.veneto.it

**Keywords:** infant, newborn, premature birth, COVID-19, interrupted time series analysis

## Abstract

Background: Nationwide studies described a reduction in preterm birth (PTB) during the Coronavirus disease 19 (COVID-19) pandemic, but this was not confirmed in others. Very few data are available on the trend of PTBs over a longer period, including the post-pandemic period, and on potential risk factors, especially those associated with social disparities. Objective: To investigate the trend in PTB rates and the influence of maternal risk factors in Italy from January 2018 to December 2022, based on 12 Regional Birth Registries covering 86.1% of Italian births. Methods: PTB trend was investigated by an interrupted time series analysis. We assessed the associations of potential risk factors with PTB (Poisson regressions) and calculated their population impact fractions (PIFs). Results: We studied 1,762,422 births; 7.25% were PTB. Before the pandemic, we observed a monthly decrease in PTB rate [−0.2% (95% CI: −0.3; −0.1)]; from July 2020 onwards, the average monthly level of PTBs was 5% lower than before (95% CI: −7.3; −2.7), with a flat trend until December 2022 [−0.02% (95% CI: −0.5; 0.5)]. Socio-economic maternal risk factors (low education, unemployment) and foreign nationality, whose risk ratios were 1.14 (95% CI: 1.04; 1.24), 1.08 (1.05; 1.12), and 1.17 (1.14; 1.21), respectively, showed a decreasing trend after pandemic; their impact on the decrease in PTBs was modest (PIFs between −2.4‰ and −4.2‰). Conclusions: The COVID-19 pandemic changed the pattern of PTB rates in Italy, lowering their average frequency and interrupting a previous decreasing trend. Changes in the trend of socio-economic maternal risk factors marginally explained this pattern.

## 1. Introduction

Preterm birth (PTB) is a major determinant of neonatal health: it is the leading cause of neonatal mortality and is associated with both short- and long-term effects, with an impact on human capital through the whole life course [1].

Historically, pregnant women exposed to crises such as wars, pandemics, or economic depressions experienced worse pregnancy outcomes, including higher rates of premature newborns, low birth weight infants, stillbirths, and neonatal mortality [2,3].

The full impact of the Coronavirus disease 19 (COVID-19) pandemic on pregnancies in the general population is still unclear. Recent nationwide studies in European countries [4,5,6,7,8], Israel [9], Japan [10], and the United States [11] found a reduction in PTBs; this was not confirmed in Scandinavian countries [12] and in Switzerland [13].

Very few data are available on the trend of PTBs after the COVID-19 pandemic [13,14]. Furthermore, investigation into how changes in time of births from mothers with risk factors for PTB might have contributed to the changing frequency of preterm birth has not been addressed yet. This could be relevant especially for socio-economic factors: during crisis more vulnerable women might have a different behavior including fertility plans.

In the present study, we aimed at:

- Investigating the trend in PTB rates in Italy—a large country and the first Western one to experience a widespread COVID-19 outbreak—focusing on the trend before, during, and after the pandemic with a longer follow-up, and using a more flexible and refined analytical strategy than in the previous study [5];
- Studying the trend of potential risk factors for PTB with information available in birth certificates within the same time frame, namely maternal age, nationality, parity, maternal education, maternal employment, and use of assisted reproductive techniques (ART) to conceive [15,16,17,18], and their impact on PTB rates.

## 2. Methods

We analyzed data from the Regional Birth Registries based on birth certificates (CeDAP, Certificato di Assistenza al Parto) which are filled in at birth for each delivery.

The study area included ten regions and two autonomous provinces (Piedmont, Lombardy, Veneto, Emilia-Romagna, Friuli-Venezia Giulia, and the Provinces of Bolzano and Trento in Northern Italy; Tuscany and Lazio in Central Italy; Apulia, Campania, and Sicily in Southern Italy) covering 86.11% of all the births occurring in Italy during the study period [19].

We collected the monthly number of live births and PTBs [live births between 22 and <37 weeks of gestational age (GA)], from 2018 to 2022. GA at birth was calculated in completed weeks and was determined based on the last menstrual period or early ultrasound scans if the last menstrual period was unknown or there was inconsistency between the two. Monthly PTB rates were computed as the ratio between PTB and all the live births.

We also collected the monthly prevalence of neonates born from mothers with the following putative risk factors for PTB: age ≥ 35 years at index birth, foreign-born women, primiparous, mothers with a low maternal education level (none or primary school or lower secondary school diploma), unemployed women (including those not in the labor force, e.g., housewives), and mothers who conceived with ART.

**Statistical analysis**: We first analyzed the log-transformed monthly trend of PTB rates (all regions combined) with an interrupted time series analysis model [20], considering seasonality and looking for a possible structural break (the interruption) [21], which was found in July 2020.

We ran a fixed-effects regression model for panels (i.e., regions) with cluster–robust standard errors using the xtreg procedure of Stata [22], once again following an interrupted time series approach with a break in July 2020 and accounting for seasonality. This model estimates pre- and post-interruption trends and a drop in the PTB rate at the interruption, while allowing different baseline PTB rates among regions/provinces and weighing estimates according to the average number of births in each region/province.

To check the fit of the model, we repeated the interrupted time series analyses separately for each region/province, considering seasonality, to verify that the trend pattern was the same in all regions. The combined estimates of change at the interruption, as well as the pre- and post-interruption, trend of each region/province were achieved with a random effect meta-analysis [23].

For the second aim, we described the trend of the prevalence of the six putative risk factors, and for each one we calculated the population attributable fraction (PAF) and the population impact fraction (PIF) [24,25]. PAF is the proportion of PTBs that would not have occurred had the risk factor been set to zero: PAF = Prevalence × (RR − 1)/[Prevalence × (RR − 1) +1]. PIF is the proportion of PTBs attributable to the decrease (or increase) in the prevalence of the risk factor in the population between two points in time; PIF = [(Prevalence_time1_ − Prevalence_time2_) × (RR − 1)]/[Prevalence_time1_ × (RR − 1) + 1].

Therefore, PAF and PIF depend on the prevalence of the risk factors and the strength of the associations between risk factors and outcome.

The association of putative PTB risk factors with PTB rates was studied in each region/province using individual data and employing multivariable Poisson regression models with robust standard error to compute mutually adjusted RR and their 95% CI for the entire period. The analyses were also adjusted for the month of birth. We then obtained an overall estimate of each factor’s RR, which was to be used in subsequent calculations of PAF and PIF, with a random effect meta-analysis.

For each factor, we computed average PAFs based on the prevalence of risk factors in two epochs, one pre-pandemic from 1 January 2018 to 28 February 2020 (before the COVID-19 restrictions were put in place) and one from 1 December 2020 to 31 December 2022. We also based computation on the RR estimates from the whole period so that changes in PAFs can be attributed only to changes in the prevalence of risk factors. We excluded births occurring within the first 9 months of COVID-19 restrictions to avoid studying risk factors in mothers of neonates born during but conceived before the pandemic. PIF was computed comparing the two epochs. We also estimated the PAF for the joint effect of the risk factors [26], which is expected to be less than the sum of the PAF for each exposure because some mothers were exposed to more than one risk factor (e.g., low education and unemployment).

All statistical analyses were carried out using Stata 15.1 (College Station, TX, USA).

Statistical analyses on individual data were run within each region. Meta-analyses were performed centrally at the Regional Health Agency of Tuscany.

## 3. Results

We studied a total of 1,762,422 live births; of these, 127,750 (7.25%) were PTBs.

**Figure 1:**
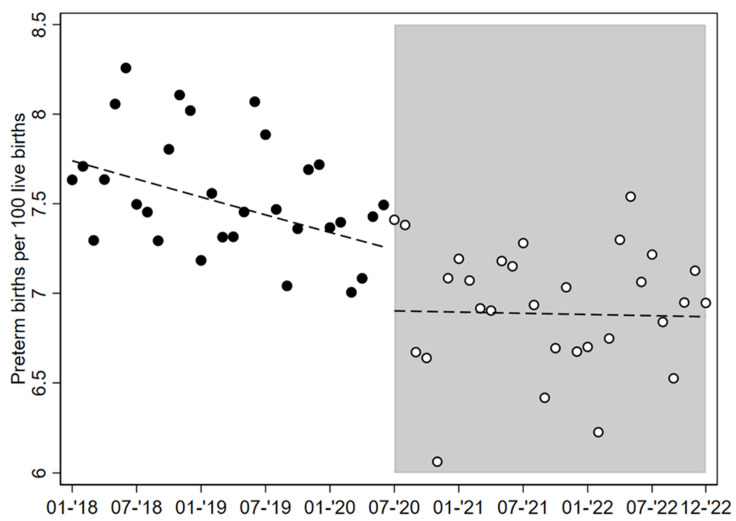
Fixed-effect regression model. Dots represent rates of preterm birth by calendar month and year from January 2018 to December 2022. Shaded area starts after the structural break in July 2020. Lines show estimated linear trends before and after the structural break.

The main model (fixed-effects regression model) (Figure 1) showed a decreasing trend in the overall rate of PTBs until the structural break found in July 2020: the trend (estimated relative change of PTB percentage) was equal to −0.2% each month (trend coefficient −0.002, 95% CI −0.003; −0.001). From July 2020 onwards, the model estimated a drop in average monthly PTB rates of −5% (coefficient −0.050, 95% CI −0.073; −0.027), corresponding to a decline of PTBs a few months after the onset of the pandemic; the trend in PTB rates remained flat until December 2022 (post-interruption trend −0.0002, 95% CI −0.005; 0.005), though visually with great oscillations.

The results of the interrupted time series regression analysis for each region are reported in Figure S1A–C. Results were very similar in different regions, except for Campania, where the post-interruption trend was negative (−0.015, 95% CI −0.019; −0.010), i.e., the PTB rates were still decreasing.

The metanalysis of regional ITSA models yielded almost identical results as the panel regression model. A comparison of the results is summarized in Table S1.

A drop in PTB rates a few months after the onset of the pandemic was also observed in very preterm (<32 weeks GA) and extremely preterm (<28 weeks GA) infants (Figure S2A,B).

**Figure 2:**
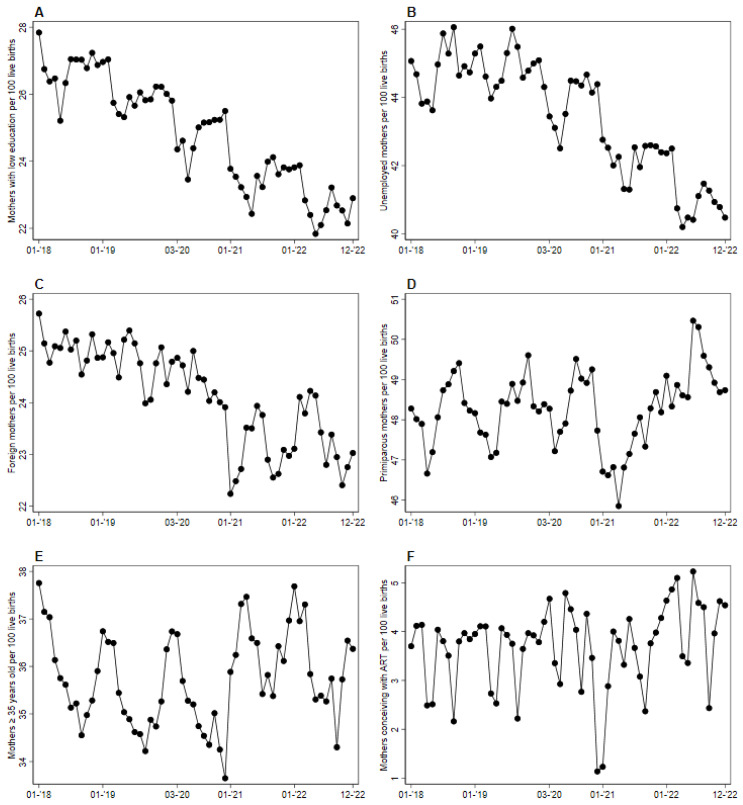
(**A**–**F**). Prevalence of putative risk factors for PTB in the population of mothers giving birth to live newborns from 1 January 2018 to 31 December 2022. (**A**) Mothers with low education per 100 live births. (**B**) Unemployed mothers per 100 live births. (**C**) Foreign mothers per 100 live births. (**D**) Primiparous mothers per 100 live births. (**E**) Mothers ≥35 years old per 100 live births. (**F**) Mothers who conceived with ART per 100 live births.

The trends of putative risk factors rates for PTB in mothers of neonates born between January 2018 and December 2022 did not vary homogeneously. A decreasing trend of births from low-educated and foreign mothers was already present before March 2020 and worsened during the pandemic, while births from unemployed mothers only decreased after a few months into the pandemic (Figure 2A–C). On the other hand, births from primiparous mothers, from mothers ≥35 years old, and from those who conceived with ART showed many short-term oscillations, with a small increase in the second epoch (for mothers >35 and who conceived by ART) but an overall flat long-term trend. (Figure 2D–F).

The associations of different risk factors with the outcome (PTB) were very similar across regions (Figure S3A–F), and the meta-analysis showed that all the putative risk factors considered were confirmed as such: mothers of low education vs. those with medium-high education (RR 1.14, 95% CI: 1.04; 1.24), mothers unemployed vs. employed (RR 1.08, 95% CI: 1.05; 1.12), foreign mothers vs. Italian (RR 1.17, 95% CI: 1.14; 1.21), primiparous vs. pluriparous (RR 1.09, 95% CI: 1.00; 1.18), mothers ≥35 years old at index birth vs. younger (RR 1.28, 95% CI: 1.22; 1.35), and mothers who conceived with ART vs. mothers who conceived spontaneously (RR 2.98, 95% CI: 2.64; 3.36).

Looking at the risk factors one by one, the PAF (percentage of PTBs that would not have occurred were the risk factor absent) did not change substantially in the 2 periods: it was around 3% for low education and unemployment, around 4% for foreign nationality and being primiparous, and—depending on higher RR—it was 9% for maternal age at child birth ≥35 years and 7% for pregnancy conceived with ART (Table 1).

The change in the trend of risk factors yielded a very small impact on PTB rates: a reduction of 0.42% (4.2 per 1000 PTBs) was attributable to the reduction in the proportion of births from low-educated mothers, of 0.24% to the reduction in the proportion of unemployed mothers, and of 0.29% to the reduction of foreign mothers. On the other hand, an increase attributable to maternal age at birth ≥35 years and to the use of ART was observed (0.12% and 0.29%, respectively).

Overall, combining the PAFs, the six risk factors accounted for 27% of PTB rates in both periods. Considering sociodemographic characteristics only (low education, unemployment) and foreign nationality, the combined PAF was 11% in the first period and 10% in the second.

## 4. Discussion

In this study, we found a decrease in the trend of PTB rates even before COVID-19 restriction measures were put in place (March 2020), which ended a few months into the pandemic (July 2020). In the subsequent months, PTB rates remained substantially stable (without increasing or decreasing trends), though at a 5% lower level than before, until December 2022. There was an association between maternal low education, unemployment, foreign nationality, primiparity, age at birth ≥35 years, and ART conception and PTB rates. These factors explained 27% of PTB rates. Socio-economic maternal risk factors (low education, unemployment) and foreign nationality showed a decreasing trend after the pandemic which, for low education and unemployment, was already partly present in the years prior; they explained 10% of PTB rates but their impact on the decrease in PTBs was modest (PIFs between −2.4‰ and −4.2‰).

The decrease in PTBs we observed before COVID-19 restrictions and up to July 2020 confirms and extends the results of the last European Perinatal Health Report [27]. The results were also based on routinely collected national data but were reported only as average yearly prevalences. According to this report, rates of PTB in Europe slightly decreased from 2015 to 2019, albeit with marked differences between countries. For Italy, the average yearly prevalence of PTBs diminished from 7.6% to 7.5%.

In our previous study [5,28], using a less refined analytical model and a much shorter follow-up, we estimated values very similar to the present ones for the pre-pandemic negative PTB trend, along with a 4.2% drop following the lockdown and mitigation strategies. The decrease in PTB rates was not accompanied by an increase in stillbirths and was similar for the subclass of late PTBs (32–36 weeks’ GA), but not for very or extremely PTBs, which had much lower frequencies and more scattered data [5]. In the present study, the drop was also similar for very and extremely PTBs.

The decrease in PTB rates that we confirmed in Italy during the COVID-19 pandemic aligns with findings from several, but not all, nationwide studies in European countries [4,5,6,7,8,12,13]. Inconsistencies in the results may reflect heterogeneity in mitigation measures and differing population characteristics and might suggest differences in the effects of lockdown [13]. We can speculate that in Italy, as well as in other Southern and Western European countries, as opposed to Scandinavian ones, the pandemic made a huge difference because of the compulsory isolation in which pregnant women (along with everyone else) were forced to live. This led to more rest, less stress at work, fewer public outings, better hygiene practices, and less exposure to other respiratory pathogens which may have contributed to the reduction in PTB.

We report a diminished number of births from mothers with low education, unemployed, and of foreign origin a few months after the start of the pandemic; nevertheless, the decrease in births of socio-economically disadvantaged and foreign mothers following COVID-19 restrictions was small, thus having a minimal impact on the observed decrease of PTBs (between 2.4 and 4.2 per 1000). A recent publication of the Euro-Peristat network showed that PTBs declined by an average 4% in 2020 in twenty-one countries compared to the previous 5 years, with no differences in socio-economic status [8].

PTB rates trends extending beyond the end of the COVID-19 pandemic have been scarcely reported so far. In Switzerland, PTB had a small but continuous downward trend through time [13]. Conversely, in the US the rate rose an average of 2% annually from 2014 to 2019, declining 1% in 2020, and then increasing again up to December 2022 [14].

PTB is a strong predictor of short- and long-term morbidity and early mortality [1]. Understanding how PTB rates vary over time in different countries is crucial for public health and healthcare organization. This can only be achieved with routinely collected data. The pandemic period and the accompanying restriction measures represented a large ‘natural experiment’ with various health implications that could possibly differ across countries. Documenting the PTB “history” in this context can provide valuable insights into PTB prevention, a field that remains largely unexplored.

Our population-based study is one of the largest carried out regarding the number and trends of PTBs over the last few years. The period analyzed allowed us to study infants born to women who were exposed to confinement and other mitigation strategies throughout their whole pregnancy.

New in this manuscript is also the analysis on risk factors for PTBs available from CeDAP and collected at birth, and the investigation on how their changes in time might have contributed to the changing frequency of PTBs.

As for limitations: we were not able to distinguish between medically indicated preterm birth or spontaneous preterm birth. Also, the dataset used does not contain information on lifestyle and social behavior of pregnant women, which precludes an analysis of possible causes raised in the literature for the observed decrease of PTBs during the COVID-19 period [4,5,9]; likewise, information on diseases in pregnancy and possible changes in health services all over the country was not available. Nonetheless, in Italy, medical consultations and care during pregnancy are free for all women, and a survey conducted in 2020 on Italian and foreign women giving birth in Northern, Central and Southern Italy showed that women attended antenatal care planned visits in more than 95% of cases [29].

Another limitation is that our study was retrospective and used routinely collected data, which are prone to registration errors, although data are filled in by midwives and physicians soon after birth and annually checked by the Ministry of Health.

## 5. Conclusions

In this study, we confirmed a decrease in PTB rates during the COVID-19 pandemic, which was not reversed after the pandemic, at least until December 2022. The decrease in births to socio-economically disadvantaged and foreign mothers following COVID-19 restrictions had a minimal impact on the observed decrease of PTBs.

## Figures and Tables

**Table 1:** Impact of maternal risk factors on preterm births: risk ratios (RR), percent population attributable fraction (PAF), and percent population impact fraction (PIF).

Maternal Risk Factors	RR (95% CI)	1 January 2018–28 February 2020		1 December 2020–31 December 2022		PIF (%)
		Prevalence (%)	PAF (95% CI) (%)	Prevalence (%)	PAF (95% CI) (%)	
Low education	1.14 (1.04–1.24)	26.36	3.49 (1.04–5.95)	23.22	3.08 (0.92–5.28)	−0.42
Unemployment	1.08 (1.05–1.12)	44.88	3.51 (2.19–5.11)	41.76	3.27 (2.05–4.77)	−0.24
Foreign nationality	1.17 (1.14–1.21)	24.92	4.11 (3.37–4.97)	23.19	3.84 (3.14–4.64)	−0.29
Primiparity	1.09 (1.00–1.18)	48.28	3.94 (0.00–8.00)	48.24	3.94 (0.00–7.99)	−0.00
Age at childbirth ≥35 years	1.28 (1.22–1.35)	35.63	9.16 (7.27–11.09)	36.07	9.26 (7.35–11.21)	0.12
Conception with ART	2.98 (2.64–3.36)	3.57	6.61 (5.54–7.78)	3.73	6.88 (5.77–8.10)	0.29

## Data Availability

Aggregated data presented in this study are available on request from the corresponding author. Restrictions apply to the availability of individual data for which analyses were run in each Region; these data are available on request from the corresponding author with the permission of the Regional Health Services.

## Supplementary Materials

The following supporting information can be downloaded at: https://www.mdpi.com/article/10.3390/children12030257/s1. Figure S1: A–C: Forest plots of the results of the interrupted time series regression analysis for each region/province and overall. (A) Pre-interruption trend (January 2018–July 2020): monthly change; (B) Change at the interruption break: July 2020 (C) Post-interruption trend (August 2020–December 2022): monthly change region-specific and overall results and 95% CI are shown: I2 = percentage of between-studies heterogeneity and relative P value. % Weight = set of weights attributed to each region/province. Figure S2: A,B: Fixed-effect regression model. Dots represent rates of preterm birth by calendar month and year from January 2018 to December 2022. Shaded area starts after structural break in July 2020. Lines show estimated linear trends before and after the structural break. (A) Very preterm infants (<32 weeks GA; n° = 15,928); from July 2020 onwards, the model estimated a drop in average monthly PTB rates of −13% (coefficient −0.125, 95% CI −0.231; −0.020); (B) Extremely preterm infants (<28 weeks GA; n° = 5,130); from July 2020 onwards, the model estimated a drop in average monthly PTB rates of −19% (coefficient −0.194, 95% CI −0.321; −0.067). Figure S3: A–F: Risk Ratios (RR) for PTB and 95% confidence interval (CI) estimated by multivariable Poisson regression models adjusted for the month of birth in different regions/provinces and combined with a random effect meta-analysis. (A) Mothers with low education vs. those with medium-high education; (B) unemployed mothers vs. employed; (C) foreign mothers vs. Italian; (D) primiparous mothers vs. pluriparous; (E) mothers ≥ 35 years old at index birth vs. youngers; (F) mothers who conceived with ART vs. mothers who conceived spontaneously. Region-specific and overall results and 95% CI are shown: I2 = percentage of between-studies heterogeneity and relative *p* value. % Weight = set of weights attributed to each region/province. Table S1: Results obtained by fixed effects regression models for panels (regions) and meta-analysis of different regions interrupted time series (ITSA) models.

## Abbreviations

The following abbreviations are used in this manuscript:

PTB	preterm birth
COVID-19	Coronavirus disease 19
CeDAP	Certificato di Assistenza al Parto
GA	gestational age
ART	assisted reproductive technique
PAF	population attributable fraction
PIF	population impact fraction
