# Supplementary material for: Trends in Preterm Births in Italy and Maternal Risk Factors in 2018–2022—A Registry-Based Study
Source: Children (Basel). 2025 Feb 20;12(3):257. doi: 10.3390/children12030257 (PMC11941680; doi:10.3390/children12030257)
Supplement: Supplementary file 1 [file children-12-00257-s001.zip › Figures S1-S3.pdf]

Figures S1 A-C. Forest plots of the results of the interrupted time series regression analysis for each region/province and overall.

(A) Pre-interruption trend (January 2018 -July 2020): monthly change

(B) Change at the interruption break: July 2020

(C) Post-interruption trend (August 2020-December 2022): monthly change

Region-specific and overall results and 95% CI are shown: I^2 = percentage of between-studies heterogeneity and relative *P* value. % Weight = set of weights attributed to each region/province.

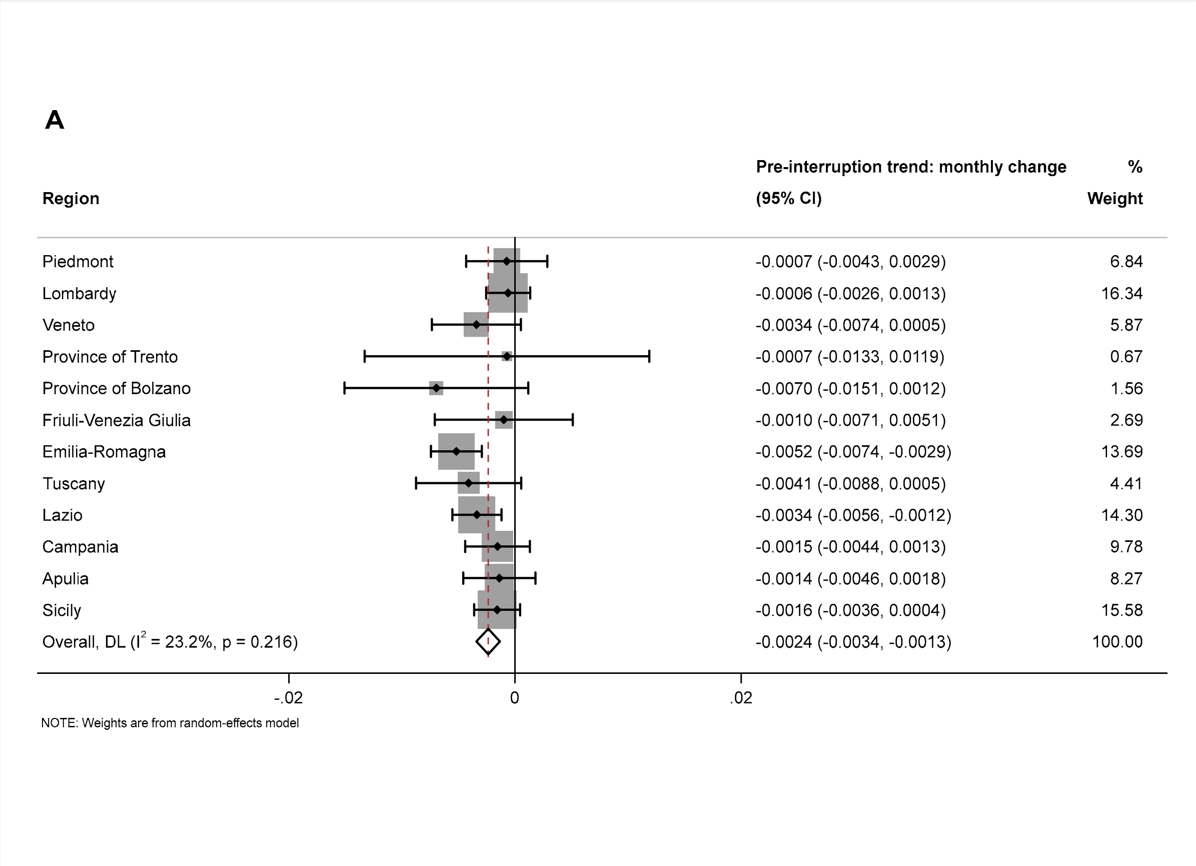

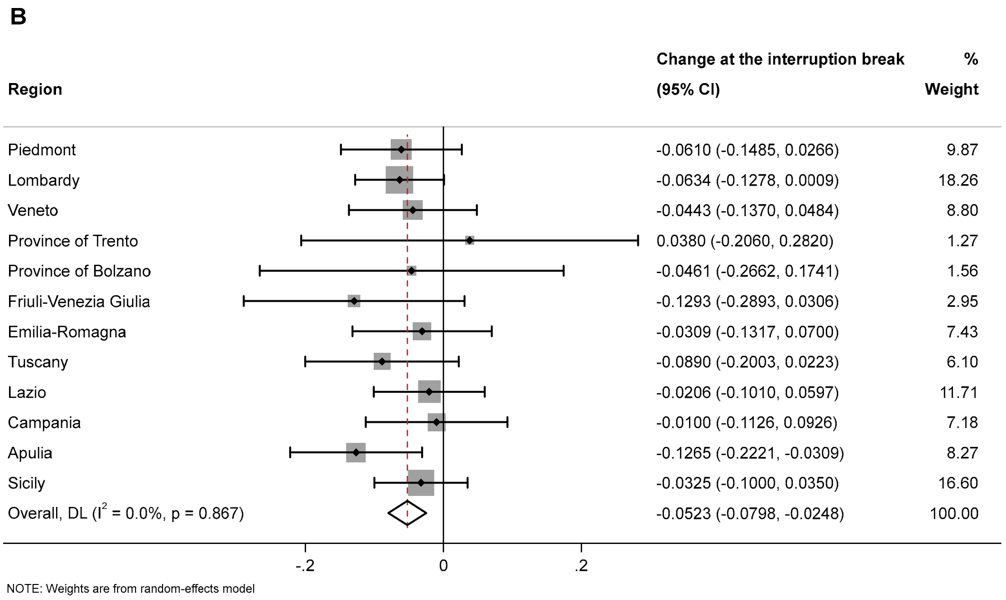
B
Region
Change at the interruption break
(95% CI)
%
Weight
Piedmont -0.0610 (-0.1485, 0.0266) 9.87
Lombardy -0.0634 (-0.1278, 0.0009) 18.26
Veneto -0.0443 (-0.1370, 0.0484) 8.80
Province of Trento 0.0380 (-0.2060, 0.2820) 1.27
Province of Bolzano -0.0461 (-0.2662, 0.1741) 1.56
Friuli-Venezia Giulia -0.1293 (-0.2893, 0.0306) 2.95
Emilia-Romagna -0.0309 (-0.1317, 0.0700) 7.43
Tuscany -0.0890 (-0.2003, 0.0223) 6.10
Lazio -0.0206 (-0.1010, 0.0597) 11.71
Campania -0.0100 (-0.1126, 0.0926) 7.18
Apulia -0.1265 (-0.2221, -0.0309) 8.27
Sicily -0.0325 (-0.1000, 0.0350) 16.60
Overall, DL (I² = 0.0%, p = 0.867) -0.0523 (-0.0798, -0.0248) 100.00
-.2
0
.2
NOTE: Weights are from random-effects model

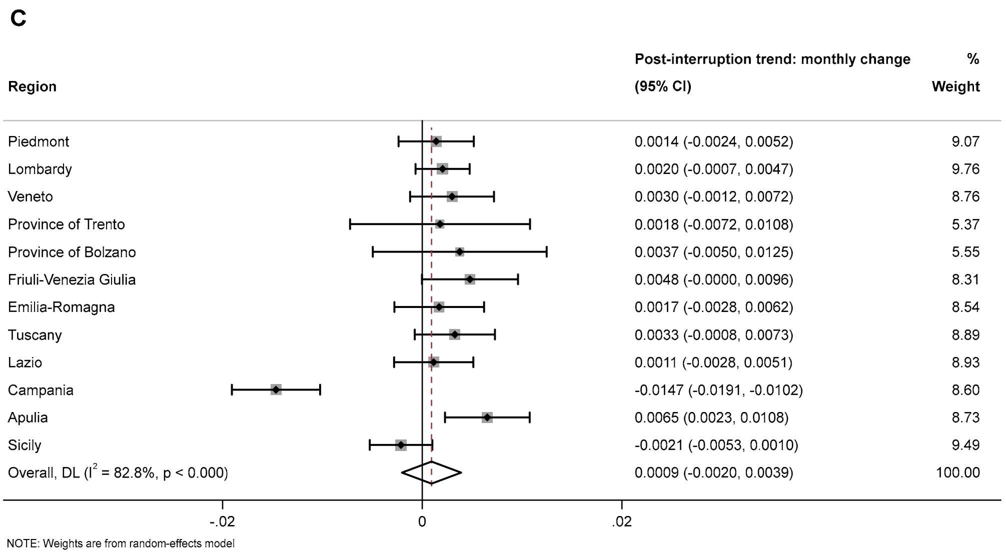
C
Region
Post-interruption trend: monthly change
(95% CI)
%
Weight
Piedmont 0.0014 (-0.0024, 0.0052) 9.07
Lombardy 0.0020 (-0.0007, 0.0047) 9.76
Veneto 0.0030 (-0.0012, 0.0072) 8.76
Province of Trento 0.0018 (-0.0072, 0.0108) 5.37
Province of Bolzano 0.0037 (-0.0050, 0.0125) 5.55
Friuli-Venezia Giulia 0.0048 (-0.0000, 0.0096) 8.31
Emilia-Romagna 0.0017 (-0.0028, 0.0062) 8.54
Tuscany 0.0033 (-0.0008, 0.0073) 8.89
Lazio 0.0011 (-0.0028, 0.0051) 8.93
Campania -0.0147 (-0.0191, -0.0102) 8.60
Apulia 0.0065 (0.0023, 0.0108) 8.73
Sicily -0.0021 (-0.0053, 0.0010) 9.49
Overall, DL (I² = 82.8%, p < 0.000) 0.0009 (-0.0020, 0.0039) 100.00
-.02
0
.02
NOTE: Weights are from random-effects model

Figures S2 A, B: Fixed-effect regression model. Dots represent rates of preterm birth by calendar month and year from January 2018 to December 2022. Shaded area starts after structural break in July 2020. Lines show estimated linear trends before and after the structural break.

(A) Very preterm infants (< 32 weeks GA; n° = 15,928); from July 2020 onwards, the model estimated a drop in average monthly PTB rates of -13% (coefficient -0.125, 95% CI -0.231; -0.020);

(B) Extremely preterm infants (< 28 weeks GA; n°= 5130); from July 2020 onwards, the model estimated a drop in average monthly PTB rates of -19% (coefficient -0,194, 95% CI -0,321;-0,067).

A

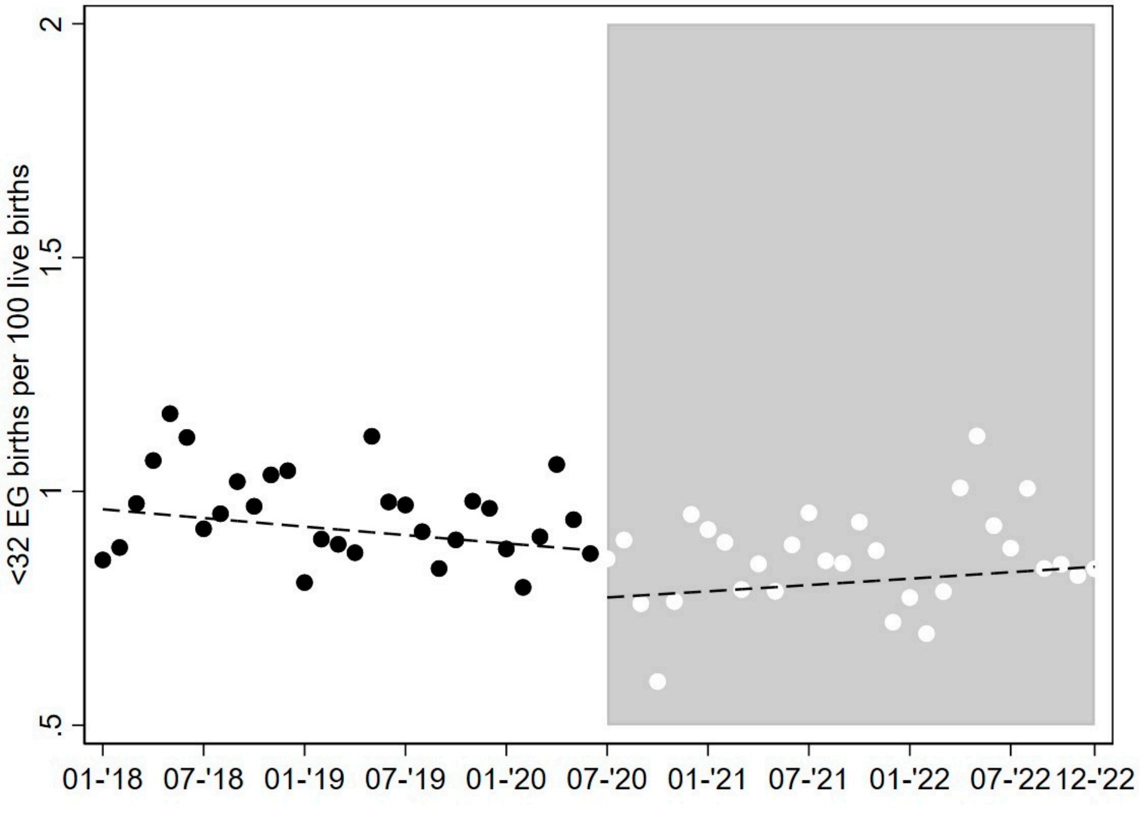

B

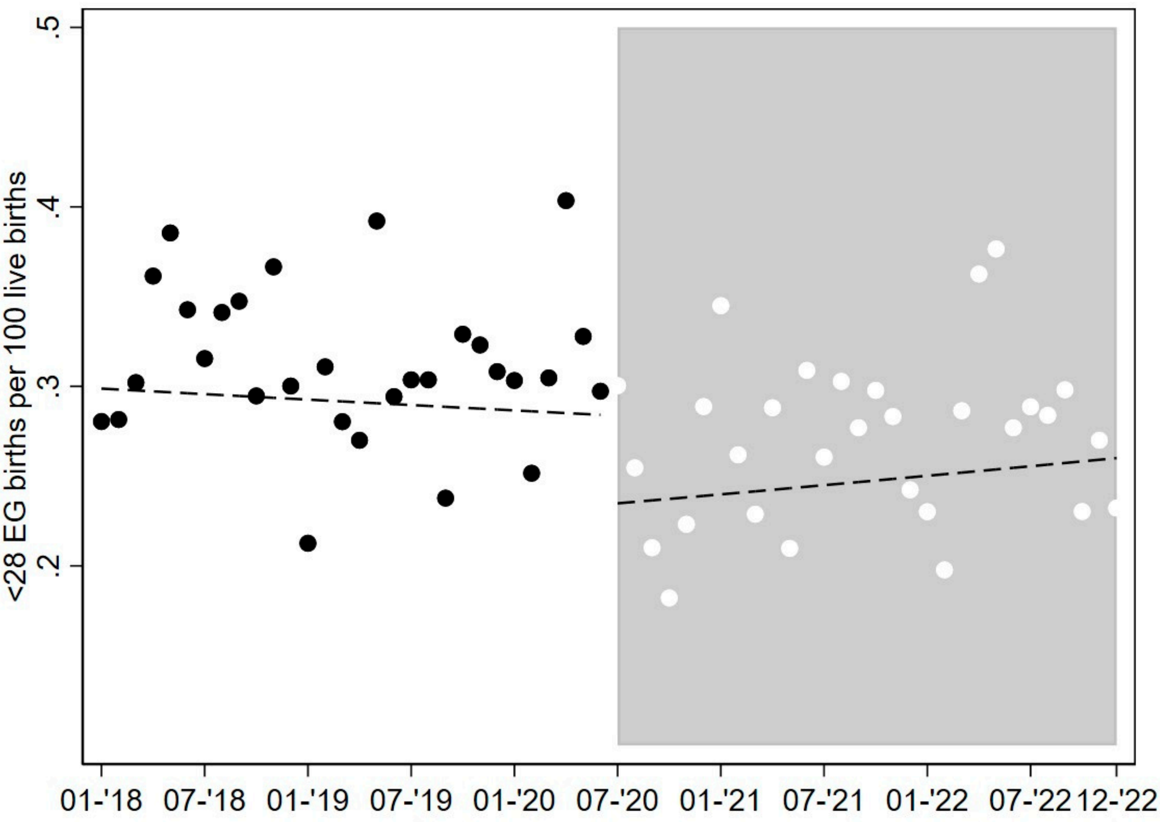

<28 EG births per 100 live births
.5
.4
.3
.2
01-'18
07-'18
01-'19
07-'19
01-'20
07-'20
01-'21
07-'21
01-'22
07-'22
12-'22

Figure S3 A-F: Risk Ratios (RR) for PTB and 95% confidence interval (CI) estimated by multivariable Poisson regression models adjusted for the month of birth in different regions/provinces and combined with a random effect meta-analysis.

A) Mothers with low education vs those with medium-high education
B) Unemployed mothers vs employed
C) Foreign mothers vs Italian
D) Primiparous mothers vs pluriparous
E) Mothers ≥ 35 years old at index birth vs youngers
F) Mothers who conceived with ART vs mothers who conceived spontaneously

Region-specific and overall results and 95% CI are shown: I^2 = percentage of between-studies heterogeneity and relative p value. % Weight = set of weights attributed to each region/province.

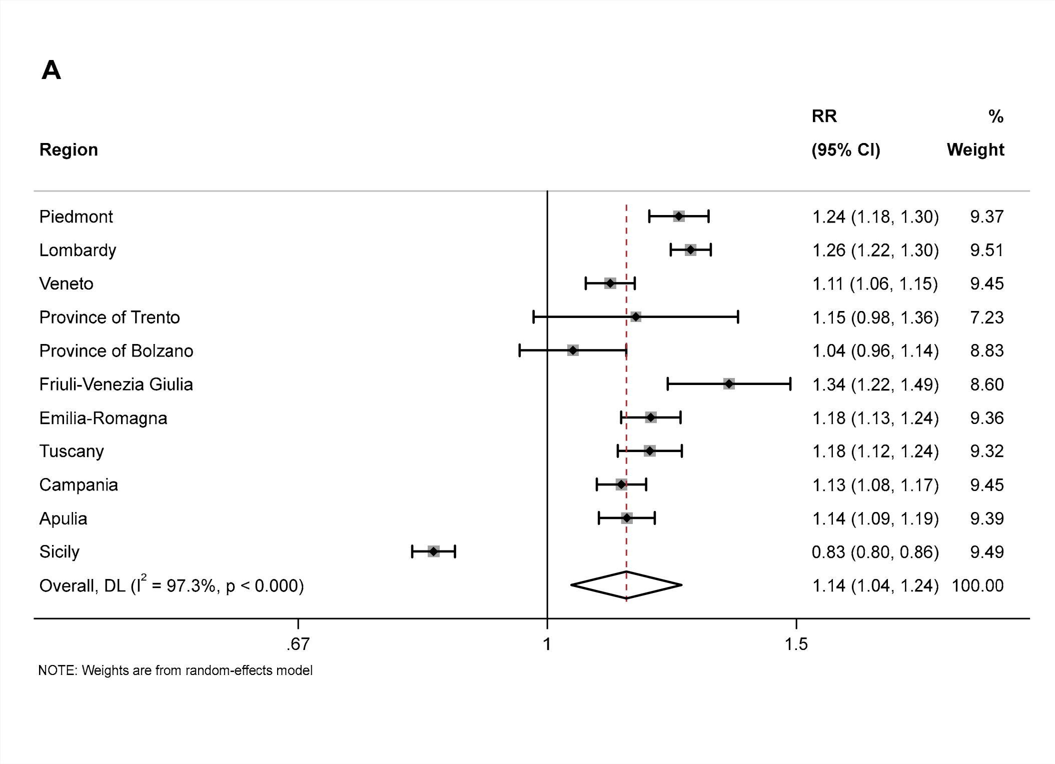

B

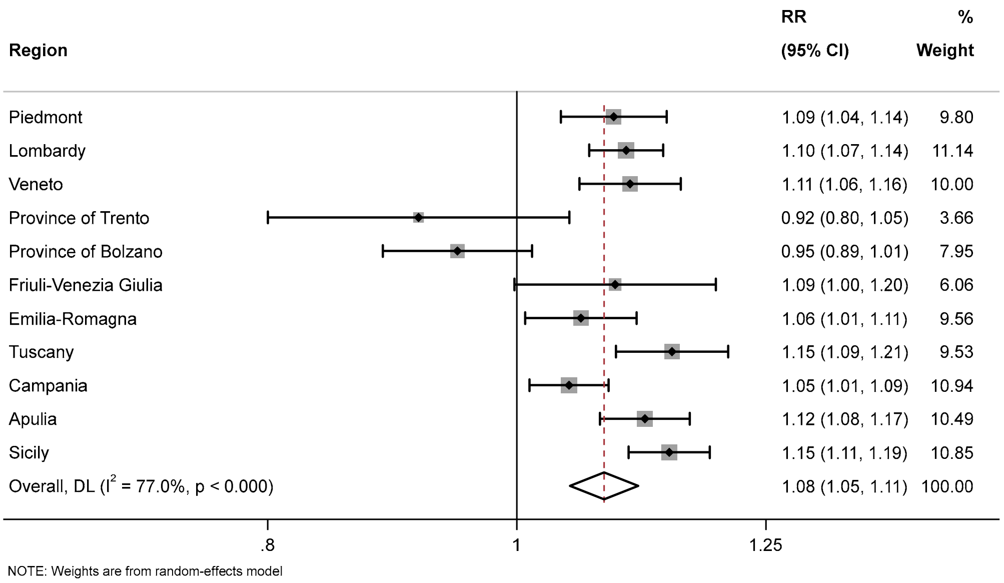
RR
%
Region
(95% CI)
Weight
Piedmont
1.09 (1.04, 1.14)
9.80
Lombardy
1.10 (1.07, 1.14)
11.14
Veneto
1.11 (1.06, 1.16)
10.00
Province of Trento
0.92 (0.80, 1.05)
3.66
Province of Bolzano
0.95 (0.89, 1.01)
7.95
Friuli-Venezia Giulia
1.09 (1.00, 1.20)
6.06
Emilia-Romagna
1.06 (1.01, 1.11)
9.56
Tuscany
1.15 (1.09, 1.21)
9.53
Campania
1.05 (1.01, 1.09)
10.94
Apulia
1.12 (1.08, 1.17)
10.49
Sicily
1.15 (1.11, 1.19)
10.85
Overall, DL (I^2 = 77.0%, p < 0.000)
1.08 (1.05, 1.11)
100.00
.8
1
1.25
NOTE: Weights are from random-effects model

C

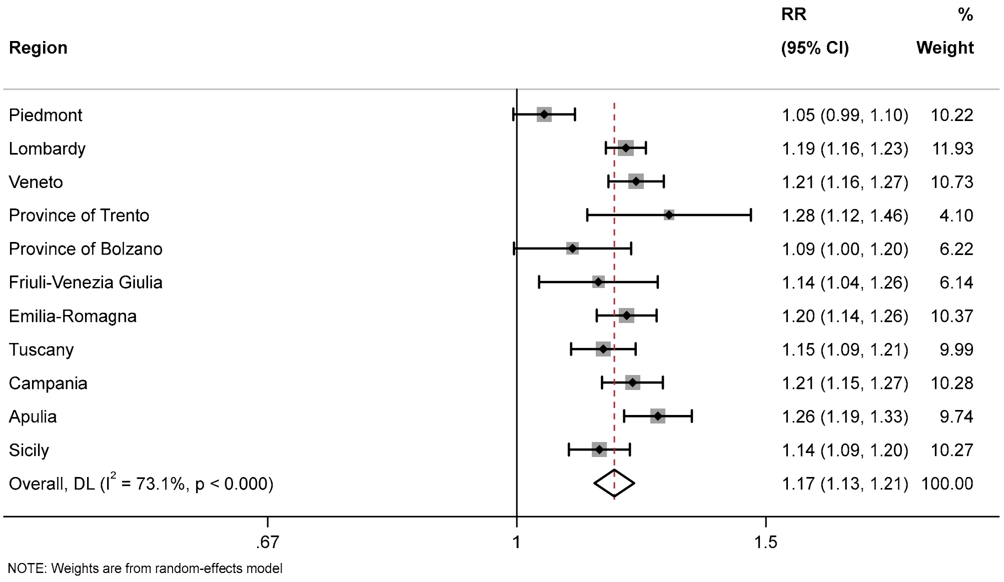
RR
%
Region
(95% CI)
Weight
Piedmont
1.05 (0.99, 1.10)
10.22
Lombardy
1.19 (1.16, 1.23)
11.93
Veneto
1.21 (1.16, 1.27)
10.73
Province of Trento
1.28 (1.12, 1.46)
4.10
Province of Bolzano
1.09 (1.00, 1.20)
6.22
Friuli-Venezia Giulia
1.14 (1.04, 1.26)
6.14
Emilia-Romagna
1.20 (1.14, 1.26)
10.37
Tuscany
1.15 (1.09, 1.21)
9.99
Campania
1.21 (1.15, 1.27)
10.28
Apulia
1.26 (1.19, 1.33)
9.74
Sicily
1.14 (1.09, 1.20)
10.27
Overall, DL (I^2 = 73.1%, p < 0.000)
1.17 (1.13, 1.21)
100.00
.67
1
1.5
NOTE: Weights are from random-effects model

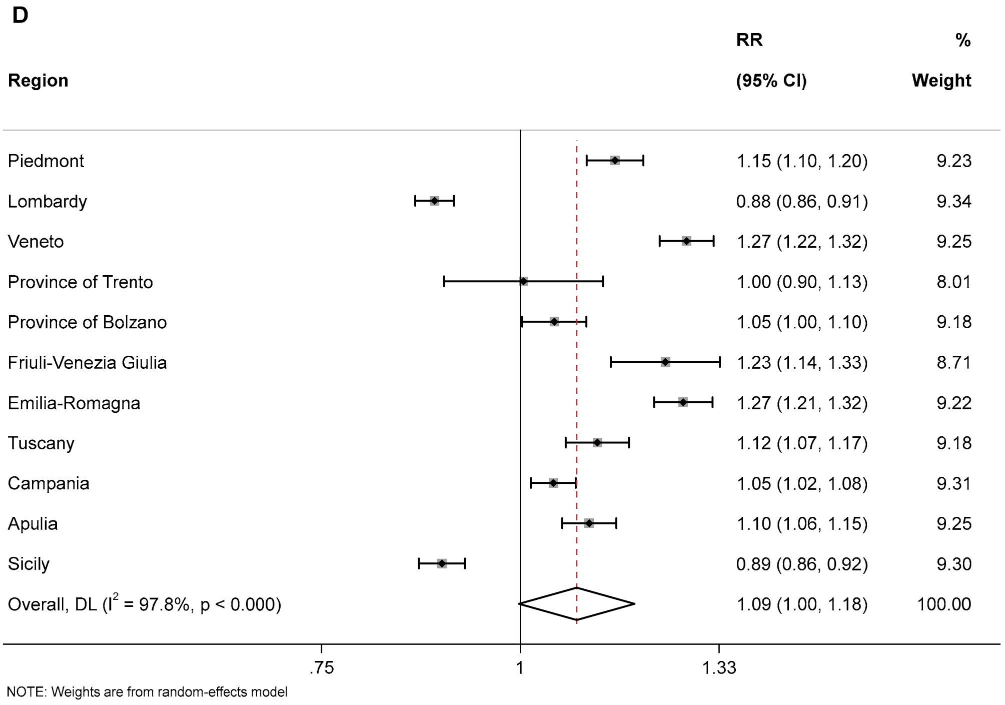
D
Region
RR
(95% CI)
%
Weight
Piedmont 1.15 (1.10, 1.20) 9.23
Lombardy 0.88 (0.86, 0.91) 9.34
Veneto 1.27 (1.22, 1.32) 9.25
Province of Trento 1.00 (0.90, 1.13) 8.01
Province of Bolzano 1.05 (1.00, 1.10) 9.18
Friuli-Venezia Giulia 1.23 (1.14, 1.33) 8.71
Emilia-Romagna 1.27 (1.21, 1.32) 9.22
Tuscany 1.12 (1.07, 1.17) 9.18
Campania 1.05 (1.02, 1.08) 9.31
Apulia 1.10 (1.06, 1.15) 9.25
Sicily 0.89 (0.86, 0.92) 9.30
Overall, DL (I2 = 97.8%, p < 0.000) 1.09 (1.00, 1.18) 100.00
.75
1
1.33
NOTE: Weights are from random-effects model

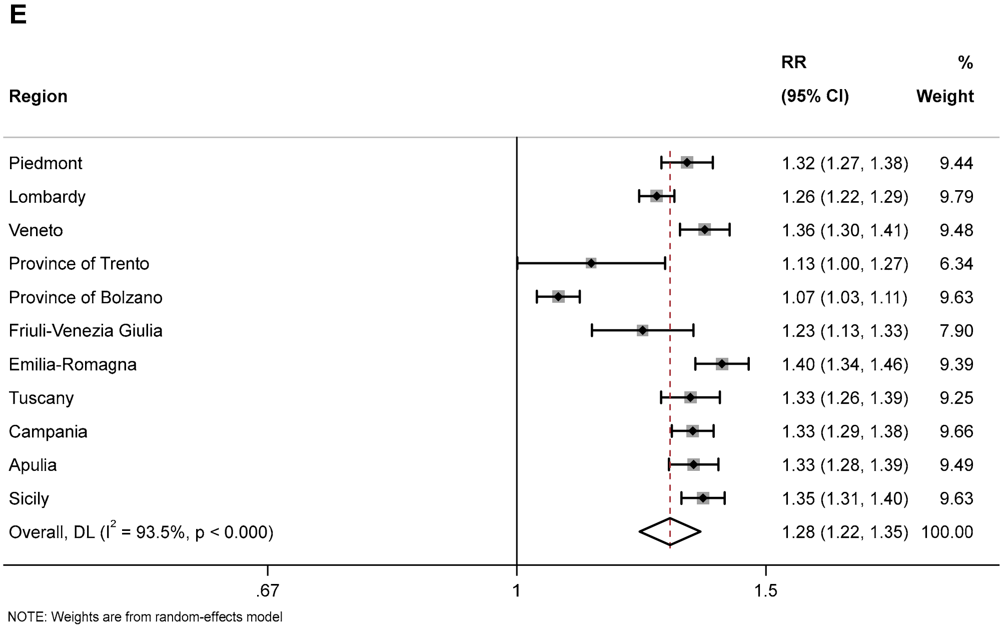
E
Region
RR
(95% CI)
%
Weight
Piedmont 1.32 (1.27, 1.38) 9.44
Lombardy 1.26 (1.22, 1.29) 9.79
Veneto 1.36 (1.30, 1.41) 9.48
Province of Trento 1.13 (1.00, 1.27) 6.34
Province of Bolzano 1.07 (1.03, 1.11) 9.63
Friuli-Venezia Giulia 1.23 (1.13, 1.33) 7.90
Emilia-Romagna 1.40 (1.34, 1.46) 9.39
Tuscany 1.33 (1.26, 1.39) 9.25
Campania 1.33 (1.29, 1.38) 9.66
Apulia 1.33 (1.28, 1.39) 9.49
Sicily 1.35 (1.31, 1.40) 9.63
Overall, DL (I2 = 93.5%, p < 0.000) 1.28 (1.22, 1.35) 100.00
.67
1
1.5
NOTE: Weights are from random-effects model

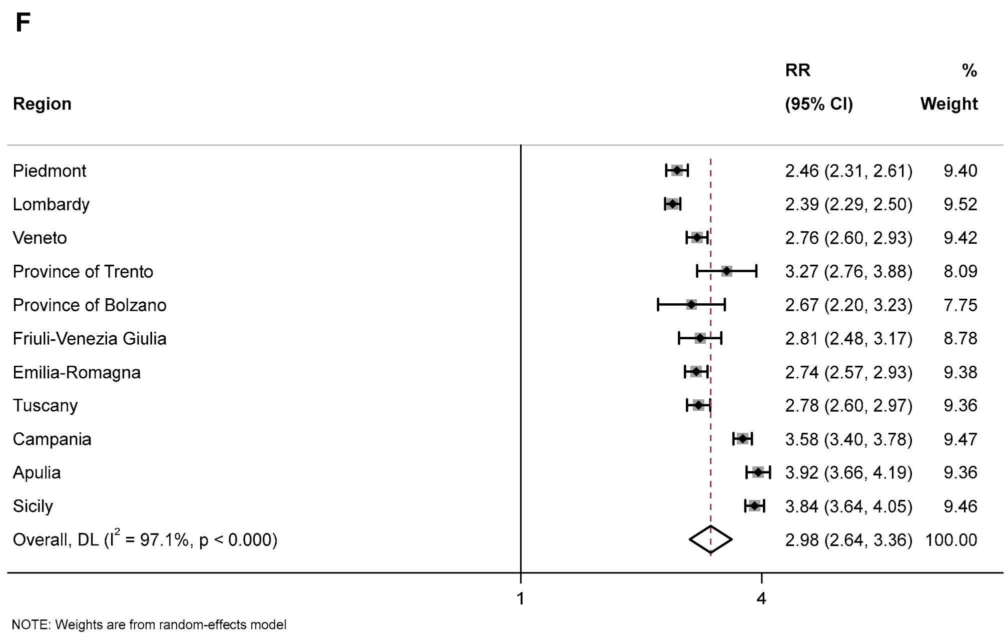
F
Region
RR
(95% CI)
%
Weight
Piedmont
2.46 (2.31, 2.61)
9.40
Lombardy
2.39 (2.29, 2.50)
9.52
Veneto
2.76 (2.60, 2.93)
9.42
Province of Trento
3.27 (2.76, 3.88)
8.09
Province of Bolzano
2.67 (2.20, 3.23)
7.75
Friuli-Venezia Giulia
2.81 (2.48, 3.17)
8.78
Emilia-Romagna
2.74 (2.57, 2.93)
9.38
Tuscany
2.78 (2.60, 2.97)
9.36
Campania
3.58 (3.40, 3.78)
9.47
Apulia
3.92 (3.66, 4.19)
9.36
Sicily
3.84 (3.64, 4.05)
9.46
Overall, DL (I^2 = 97.1%, p < 0.000)
2.98 (2.64, 3.36)
100.00
1
4
NOTE: Weights are from random-effects model
