# Supplementary material for: Trends in Preterm Births in Italy and Maternal Risk Factors in 2018–2022—A Registry-Based Study
Source: Children (Basel). 2025 Feb 20;12(3):257. doi: 10.3390/children12030257 (PMC11941680; doi:10.3390/children12030257)
Supplement: Supplementary file 1 [file children-12-00257-s001.zip › Table S1.pdf]

Table S 1. Results obtained by fixed effects regression models for panels (regions) and meta-analysis of different regions interrupted time series (ITSA) models.

	Fixed-effects panel regression	ITSA meta-analysis
Pre-interruption trend: monthly change (95% CI)	-0.0022 (-0.0034; -0.0011)	-0.0024 (-0.0034; -0.0013)
Change at the interruption, July 2020 (95% CI)	-0.0503 (-0.0733; -0.0273)	-0.0523 (-0.0798; -0.0248)
Post-interruption trend: monthly change (95% CI)	-0.0002 (-0.0048; 0.0046)	0.0009 (-0.0020; 0.0039)
